# Hepatitis B virus infection and active replication promote the formation of vascular invasion in hepatocellular carcinoma

**DOI:** 10.1186/s12885-017-3293-6

**Published:** 2017-05-02

**Authors:** Xubiao Wei, Nan Li, Shanshan Li, Jie Shi, Weixing Guo, Yaxin Zheng, Shuqun Cheng

**Affiliations:** Department of Hepatic Surgery VI, Eastern Hepatobiliary Surgery Hospital, Second Military Medical University, 225 Changhai Road, Yangpu District, Shanghai, 200438 China

**Keywords:** Hepatitis B virus, Hepatocellular carcinoma, Vascular invasion, Anti-viral treatment, Postoperative recurrence

## Abstract

**Background:**

Vascular invasion, including microvascular invasion (MVI) and portal vein tumor thrombus (PVTT), is associated with the postoperative recurrence of hepatocellular carcinoma (HCC). We aimed to investigate the potential impact of hepatitis B virus (HBV) activity on the development of vascular invasion.

**Methods:**

Patients with HBV and tumor-related factors of HCC who had undergone hepatectomy were retrospectively enrolled and analyzed to identify the risk factors for developing vascular invasion.

**Results:**

A total of 486 patients were included in this study. The overall proportion of patients with vascular invasion, including MVI and PVTT, was 60.3% (293/486). The incidence of MVI was 58.2% (283/486) whereas PVTT was 22.2% (108/486). Univariate analysis revealed that positive Hepatitis B virus surface Antigen (HBsAg) was significantly associated with the presence of vascular invasion. In a multivariate regression analysis carried out in patients with HBV-related HCC, positive Hepatitis B virus e Antigen (HBeAg)(OR = 1.83, *P* = 0.019) and a detectable seral HBV DNA load (OR = 1.68, *P* = 0.027) were independent risk factors of vascular invasion. The patients in the severe MVI group had a significantly higher rate of positive seral HBsAg (*P* = 0.005), positive seral HBeAg (*P* = 0.016), a detectable seral HBV DNA load (> 50 IU/ml) (*P* < 0.001) and a lower rate of anti-viral treatment (*P* = 0.002) compared with those in the mild MVI group and MVI-negative group. Whereas, HCC with PVTT invading the main trunk showed a significantly higher rate of positive HBsAg (*P* = 0.007), positive HBeAg (*P* = 0.04), cirrhosis (*P* = 0.005) and a lower rate of receiving antiviral treatment (*P* = 0.009) compared with patients with no PVTT or PVTT invading the ipsilateral portal vein. Patients with vascular invasion also had a significantly higher level of seral HBV DNA load than patients without vascular invasion (*P* = 0.008).

**Conclusions:**

In HCC patients, HBV infection and active HBV replication were associated with the development of vascular invasion.

## Background

Hepatocellular carcinoma (HCC) is the fifth most common cancer and the third leading cause of cancer-related death in the world [1]. Although surgical resection and liver transplantation could offer a promising prognosis for selected patients with HCC, the high postoperative recurrence rate has impaired long-time survival. Among various risk factors, vascular invasion, including microvascular and macrovascular invasion, has been proven to be an independent factor predicting high recurrence and poor survival rate [2–4]. Microvascular invasion (MVI) was defined by most studies as microscopically confirmed tumor cell clusters within a vascular cavity lined with endothelium adjacent to the tumor [5, 6]. Conversely, macrovascular invasion mostly occurs in the portal vein system and is known as a portal vein tumor thrombus (PVTT); a PVTT can be identified during imaging examination or intraoperative exploration. A large tumor size, multinodular lesion, elevated level of desc-carboxy prothrombin (DCP) and certain imaging characteristics were reported to be factors predicting the presence of MVI, whereas the tumor size, Edmondson-Steiner histological grading, number of nodules and α-fetoprotein (AFP) level were associated with PVTT [2, 5, 7, 8].

Chronic hepatitis B virus (HBV) infection is a major risk factor for the development of liver cirrhosis and HCC, especially in East Asia [6]. HBV-related factors, such as seropositivity of hepatitis B e-antigen (HBeAg), high hepatitis B surface antigen (HBsAg) level and high serum HBV DNA load, were found to be significantly related to an increased risk of HBV-associated cirrhosis and HCC [9, 10]. These factors were also reported to be associated with an increased recurrence rate and a decreased survival rate of HCC after surgical resection [11, 12]. Fundamental research has revealed that the HBV-initiated tumorigenic process may play a role in the development of the vascular invasion of HCC [13–15]. Recently, Lei Z et al. established a nomogram for preoperative prediction of the presence of MVI in HBV-related HCC, in which a high HBV DNA load (>10^4^ IU/ml) was independently associated with the development of MVI [16]. These findings indicated a potential correlation between active HBV replication and the development of vascular invasion in HCC. To the best of our knowledge, no published study has provided insight into this issue. Therefore, we conducted a clinical study to further explore the impact of HBV-related factors on the formation of vascular invasion in HCC.

## Methods

### Study population

This was a retrospective study based on a prospectively compiled clinical and pathology database at a treatment center for HCC with PVTT at the Eastern Hepatobiliary Surgery Hospital, Shanghai, China. The study was approved by our Institutional Review Board, and written informed consent was obtained from all patients for their data to be used in this research.

HCC patients who had undergone surgical resection and confirmed by pathological examination at our center were included in this study. Exclusion criteria included hepatitis C virus (HCV)-related HCC, preoperative transarterial chemoembolization (TACE) or radiotherapy, non-curative resection, recurrent lesions, and a lack of complete clinical or pathological information.

For patients included in the study, the following clinical data and pathological results were collected: (1) demographic data, including age and gender and history of anti-viral treatment; (2) results of preoperative laboratory blood tests, including HBsAg, HBeAg, HBV-DNA level, AFP, DCP, albumin, total bilirubin, alanine aminotransferase, and aspartate aminotransferase; and (3) imaging and pathologic findings, including the presence and classification of PVTT, maximal tumor size, tumor number, capsule, presence and classification of MVI, and presence of cirrhosis.

Tests for the viral replication status, including those for HBsAg and its antibody, HBeAg and its antibody, and HBcAb, were performed. The serum HBV-DNA level was quantified by the polymerase chain reaction assay (ABI 7500; Applied Biosystems, Foster City, CA, USA) with a linear range of quantification of 50 to 2,000,000 IU/ml. The lower limit of detection was 50 IU/ml. Patients who had received standard interferon therapy or had been using oral anti-viral drugs for a duration of more than 2 months before surgery were classified as the anti-viral treatment group.

### Diagnostic criteria of vascular invasion

The diagnostic criterion of MVI was the presence of a tumor cell nest in the portal vein, hepatic artery, hepatic vein, bile duct or lymph duct in the tumor surrounding the liver tissue under microscopic examination [2, 17]. The number and distribution of invaded vessels were measured to divide the patients with MVI into two groups as follows: patients in the mild MVI group (M1) had 1 to 5 involved vessels distributed within a 1-cm area from the tumor margin, whereas patients in the severe MVI group (M2) had more than 5 vessels invaded or had invaded vessels located more than 1 cm from the tumor margin. Every specimen was reviewed independently by two senior hepatobiliary pathologists to detect MVI. If the two pathologists had an inconsistent diagnosis, the findings were discussed to reach a final decision.

All HCC patients admitted to our center underwent a routine three-phrase dynamic CT or MRI examination before any treatment was carried out. PVTT was diagnosed when there were low-attenuation intraluminal masses that expanded the portal vein, or filling defects in the portal vein system, as presented in CT or MRI imaging. PVTT was confirmed and reassessed by palpation or ultrasound during operation. The final diagnosis was dependent on the intraoperative or pathologic findings. PVTT was classified according to Cheng’s classification, which has been shown to be effective in stratifying the severity of PVTT as follows: type I, invasion of the tumor thrombus into the segmental or sectoral branches of the portal vein or above; type II, involvement of the right or left portal vein; type III, invasion of the main trunk of the portal vein; and type IV, involvement of the superior mesenteric vein.

### Statistical analysis

All calculations were performed using Stata 12.0 software (StataCorp, Texas 77,845, USA). Continuous and categorized data were compared using Pearson’s chi-squared test, Fisher’s exact test, or Student’s t test, as appropriate. Binary logistic regression was used to evaluate the relationship between the presence of vascular invasion as the dependent variable and factors that were significant in the univariate analysis as independent variables, using the stepwise backward method (Wald). The enter limit and remove limit were *P* = 0.05 and *P* = 0.10, respectively. Because viral factors, including seral HbeAg, the seral HBV DNA load, presence of cirrhosis and usage of antiviral treatment, were only meaningful when patients had HBV infection, only patients with positive seral HbsAg were included in multivariate analysis. A *P* < 0.05 was considered to indicate statistical significance.

## Results

From May 1, 2015 to July 31, 2016, 675 patients with a preoperative diagnosis of HCC who underwent surgical resection at our center were identified. After careful examination, 189 patients were excluded, including 77 for preoperative TACE or radiotherapy, 36 for being diagnosed with a histological type other than HCC, 7 for HCV infection, 27 for recurrent lesions, 21 for non-curative resection, and 21 for failure to obtain detailed clinic information. Finally, 486 HCC patients, 422 men and 64 women, with a median age of 52 years (range, 22–80 years), fulfilled the inclusion criteria and were enrolled in the study.

Most patients (88.5%, 430/486) had HBV-related HCC; the remaining 56 patients (11.5%) had negative serum HBsAg. A total of 297 patients (61.1%) had a detectable seral HBV DNA load (> 50 IU/ml) in which 108 patients (22.2%) had a high HBV DNA load level > 2000 IU/ml. In total, 130 patients (26.7%) were classified into the anti-viral treatment group (interferon, 7; lamivudine, 14 patients; lamivudine + adefovir dipivoxil, 7 patients; lamivudine + entecavir, 11 patients; adefovir dipivoxil, 26 patients; entecavir, 33 patients; entecavir + adefovir dipivoxil, 8 patients; others, 24 patients). The overall proportion of patients with vascular invasion, including MVI and PVTT, was 60.3% (293/486). The incidence of MVI was 58.2% (283/486), whereas that of PVTT was 22.2% (108/486). A total of 98 patients (20.2%) had both MVI and PVTT.

### Univariate analysis of viral and tumor factors predicting vascular invasion in HCC

Univariate analysis revealed that virus-related serum markers, including positive HBsAg (*P* = 0.005), positive HBeAg (*P* < 0.001) and a detectable HBV DNA load (*P* < 0.001), were significantly associated with the presence of vascular invasion, whereas vascular invasion was less frequently detected in patients in the anti-viral drug group (*P* = 0.003). The significant viral factors predicting MVI were the same as those of vascular invasion for patients with vascular invasion and MVI and were mostly overlapping. Similarly, virus-related seral markers, including positive HBsAg (*P* = 0.003), positive HBeAg (*P* = 0.005), a detectable HBV DNA load (*P* = 0.025), and the presence of cirrhosis (*P* < 0.001), were significantly associated with the presence of PVTT, whereas patients who were undergoing anti-viral treatment (*P* = 0.015) had a significantly lower risk of developing PVTT (Table 1).

**Table 1:** Univariate analysis of risk factors for formation of vascular invasion in hepatocellular carcinoma patients who underwent hepatectomy

Parameters	Vascular invasion		*P*	MVI		*P*	PVTT		*P*
	yes	no		yes	no		yes	no	
Patient demographics									
Age									
> 50 years	153	125	0.006*	149	129	0.017*	53	225	0.053
< = 50 years	140	68		134	74		55	153	
Gender									
Male	255	167	0.873	245	177	0.842	99	323	0.092
Female	38	26		38	26		9	55	
Preoperative laboratory test									
Total bilirubin									
> 20 μmol/l	48	32	0.954	48	32	0.725	16	64	0.601
< = 20 umol/l	245	161		235	171		92	314	
ALT									
> 42 U/l	128	83	0.882	122	89	0.872	49	162	0.642
< = 42 U/l	165	110		161	114		59	216	
AST,									
> 37 U/l	157	114	0.234	152	119	0.282	62	209	0.696
< = 37 U/l	136	79		131	84		46	169	
Albumin									
> 40 g/l	193	100	0.734	188	135	0.987	67	256	0.270
< = 40 g/l	130	63		95	68		41	122	
DCP									
> 100 mAU/ml	250	147	0.011*	241	156	0.019*	102	295	<0.001*
< = 100 mAU/ml	43	46		42	47		6	83	
Alpha-fetoprotein									
> 20 ng/ml	226	102	<0.001*	219	109	<0.001*	85	243	0.005*
< = 20 ng/ml	67	91		64	94		23	135	
Tumor characteristics									
Diameter									
> 3 cm	247	138	0.001*	238	147	0.002*	105	280	<0.001*
< = 3 cm	46	55		45	56		3	98	
Number of lesions									
Multiple	43	15	0.022*	41	17	0.040*	20	38	0.017*
Single	250	178		242	186		88	340	
Encapsulation									
Incomplete/absent	259	145	<0.001*	250	154	<0.001*	101	303	0.001*
Complete	34	48		33	49		7	75	
Edmonson grading									
Grades III/IV	277	151	<0.001*	267	161	<0.001*	101	327	0.047*
Grades I/II	16	42		16	42		7	51	
Virus-related factors									
Seral HBsAg									
Positive	269	161	0.005*	260	170	0.006*	104	326	0.003*
Negative	24	32		23	33		4	52	
Seral HBeAg									
Positive	93	32	<0.001*	88	37	0.002*	39	86	0.005*
Negative	200	160		195	165		69	291	
HBV DNA load									
Detectable (> 50 IU/ml)	203	94	<0.001*	197	100	<0.001*	76	221	0.025*
Undetectable (<=50 IU/ml)	90	99		86	103		32	157	
High (> 2000 IU/ml)	110	62	0.222	106	66	0.261	39	133	0.859
Low (<= 2000 IU/ml)	183	131		177	137		69	39	
Presence of cirrhosis									
Yes	101	52	0.08	96	57	0.171	49	104	<0.001*
No	192	141		187	146		59	274	
Anti-virus treatment									
Yes	64	66	0.003*	63	67	0.008*	19	111	0.015*
No	229	127		220	136		89	267	

*MVI* Microvascular invasion; *PVTT* Portal vein tumor thrombus; *ALT* Alanine aminotransferase; *AST* Aspartate aminotransferase; *DCP* Des-gamma-carboxy prothrombin; *HBsAg* Hepatitis B virus s Antigen; *HBeAg* Hepatitis B virus e Antigen; *HBV* Hepatitis B virus

*P < 0.05

### Multivariate analysis of viral and tumor factors predicting vascular invasion in HCC

Multivariate logistic regression analysis was carried out in patients with positive seral HbsAg, utilizing binary variables that were significant in the univariate analysis. As shown in Table 2, positive seral HBeAg (OR = 1.83, *P* = 0.019) and a detectable seral HBV DNA load (OR = 1.68, *P* = 0.027) were independent risk factors of vascular invasion in the multivariate regression analysis. Moreover, tumor-related factors, including a seral AFP level > 20 ng/ml (OR = 2.51, *P* < 0.001), multiple lesions (OR = 2.18, *P* = 0.038), tumor size >3 cm (OR = 1.73, *P* = 0.035), Edmonson grades III/IV (OR = 2.48, *P* = 0.013) and incomplete/absent tumor capsule (OR = 2.17, *P* = 0.006), were significantly and independently associated with vascular invasion. Factors predictive of MVI were similar to those predictive of vascular invasion, except that the impact of seral HBeAg on the formation of MVI didn’t reach statistical significance (OR = 1.59, *P* = 0.059). Regarding the risk factors of PVTT, the impact of the seropositivity of HBeAg (OR = 1.67, *P* = 0.046), tumor diameter > 3 cm (OR = 8.86, *P* < 0.001), incomplete or absent encapsulation (OR = 3.59, *P* = 0.003) and DCP > 100 mAU/ml (OR = 2.90, *P* = 0.022) were significant in the multivariable analysis.

**Table 2:** Multivariate logistic regression analysis of factors predictive of vascular invasion in patients with positive seral HbsAg

Variables	Odds ratio	95% CI	*P* value
Risk of vascular invasion			
Age (>50 years vs < =50 years)	0.68	0.44–1.04	0.078
Alpha-fetoprotein (> 20 ng/ml vs < = 20 ng/ml)	2.51	1.59–3.96	<0.01*
Tumor number (Multiple vs Single)	2.18	1.04–4.55	0.038*
Diameter (> 3 cm vs < = 3 cm)	1.73	1.04–2.88	0.035*
Edmonson grading (Grades III/IV vs Grades I/II)	2.48	1.21–5.05	0.013*
Tumor capsule (Incomplete/absent vs Complete)	2.17	1.25–3.77	0.006*
Seral HBeAg (Positive vs Negative)	1.83	1.10–3.03	0.019*
Seral HBV DNA load (> 50 IU/ml vs < = 50 IU/ml)	1.68	1.06–2.65	0.027*
Risk of microscopic vascular invasion			
Alpha-fetoprotein (> 20 ng/ml vs < = 20 ng/ml)	2.59	1.65–4.05	<0.01*
Tumor number (Multiple vs Single)	2.21	1.09–4.51	0.028*
Diameter (> 3 cm vs < = 3 cm)	1.58	0.96–2.61	0.074
Edmonson grading (Grades III/IV vs Grades I/II)	2.24	1.11–4.54	0.024*
Tumor capsule (Incomplete/absent vs Complete)	2.04	1.18–3.51	0.011*
Seral HBeAg (Positive vs Negative)	1.59	0.98–2.57	0.059
Seral HBV DNA load (> 50 IU/ml vs < = 50 IU/ml)	1.76	1.12–2.76	0.013*
Risk of portal vein tumor thrombus			
DCP (>100 mAU/ml vs < = 100 mAU/ml)	2.90	1.17–7.12	0.022*
Tumor diameter (>3 cm vs < =3 cm)	8.86	2.67–29.39	<0.01*
Tumor capsule (Incomplete/absence vs Complete)	3.59	1.56–8.25	0.003*
Seral HBeAg (Positive vs Negative)	1.67	1.01–2.75	0.046*
Anti-virus treatment (Yes vs No)	0.59	0.33–1.05	0.075

*CI* Confidential Interval; *HBeAg* Hepatitis B virus e Antigen; *HBV* Hepatitis B virus; *DCP* Des-gamma-carboxy prothrombin; *HBsAg* Hepatitis B virus s Antigen; *HBeAg* Hepatitis B virus e Antigen

*P < 0.05

### Correlation between the features of vascular invasion and HBV-related factors

Table 3 shows that the severe MVI group had a significantly higher rate of positive seral HBsAg, positive seral HBeAg, a detectable seral HBV DNA load (> 50 IU/ml), as well as a lower rate of antiviral treatment, compared with the mild MVI group and negative group. By contrast, for the classification of PVTT, HCC with type III/IV PVTT had a significantly higher rate of positive seral HBsAg, positive seral HBeAg, and cirrhosis, as well as a lower rate of receiving antiviral treatment compared with the type I/II group and PVTT-negative group. Patients with vascular invasion had a significantly higher seral HBV DNA load than patients without vascular invasion (Table 4).

**Table 3:** Correlations between the severity of microvascular invasion or portal vein tumor thrombus and viral features in hepatocellular carcinoma^a^

HBV-related factors	Severity of MVI			*P*	Classification of PVTT			*P*
	None (%)	Mild (%)	Severe (%)		None (%)	I/II (%)	III/IV (%)	
Seral HBsAg								
Positive	171 (83.8)	127 (89.4)	131 (94.9)	0.005*	326 (86.2)	74 (94.9)	30 (100)	0.007*
Negative	33 (16.2)	15 (10.6)	7 (5.1)		52 (13.8)	4 (5.1)	0 (0)	
Seral HBeAg								
Positive	37 (21.6)	38 (29.9)	48 (36.6)	0.016*	84 (25.8)	30 (40.5)	9 (30)	0.04*
Negative	134 (78.4)	89 (60.1)	83 (63.4)		242 (74.2)	44 (59.5)	21 (70)	
Seral HBV DNA load								
> 50 IU/ml	99 (57.9)	90 (70.9)	106 (80.9)	<0.001*	219 (67.2)	52 (70.3)	24 (80)	0.355
< = 50 IU/ml)	72 (42.1)	37 (29.1)	25 (19.1)		107 (32.8)	22 (29.7)	6 (20)	
Presence of cirrhosis								
Yes	52 (30.4)	48 (37.8)	44 (33.6)	0.418	96 (29.4)	32 (43.2)	16 (53.3)	0.005*
No	119 (69.6)	79 (62.2)	87 (66.4)		230 (60.6)	42 (56.8)	14 (46.7)	
Antivirus treatment								
Yes	66 (38.6)	36 (28.3)	26 (19.8)	0.002*	109 (33.4)	15 (20.3)	4 (13.3)	0.009*
No	105 (61.4)	91 (61.7)	105 (80.2)		217 (66.6)	59 (79.7)	26 (86.7)	

*HBV* Hepatitis B virus; *HBsAg* Hepatitis B virus s Antigen; *HBeAg* Hepatitis B virus e Antigen

^a^analysis was only carried out in patients with positive HBsAg except the “HBsAg” row

*P < 0.05

**Table 4:** Difference in the seral HBV DNA load between HBV-related hepatocellular carcinoma patients with or without vascular invasion^a^

Variable	HBV DNA load, log _10_ IU/ml (mean ± SD)	*P*
Vascular invasion		
Yes	3.28 ± 0.14	0.008*
No	2.64 ± 0.20	
Microvascular invasion		
Yes	3.29 ± 0.14	0.008*
No	2.66 ± 0.19	
Portal vein tumor thrombus		
Yes	3.13 ± 0.22	0.694
No	3.02 ± 0.14	

*HBV* Hepatitis B virus; *SD* Standard Deviation

^a^analysis was only carried out in patients with positive HBsAg

*P < 0.05

## Discussion

The presence of vascular invasion, including MVI and PVTT, was significantly associated with a high risk of postoperative recurrence, which is a major obstacle to improving the prognosis of HCC [6, 17, 18]. However, the risk factors and underlying mechanism leading to the formation of vascular invasion remain largely unknown. In East Asia, the majority of HCC develops within an environment of chronic inflammation caused by HBV infection. Recently, the results of fundamental studies have indicated that the HBV status is a potent etiological factor predisposing HCC patients to develop vascular invasion. HBV X protein (HBx), a key regulatory multifunctional protein of the virus, has been reported to be involved in the development of MVI and is associated with postoperative recurrence [14, 19, 20]. Yang et al. found that the seropositivity of HBsAg was associated with a high risk of developing PVTT, and the activity of the TGF-β-miR-34a-CCL22 axis induced by the change in the liver microenvironment caused by HBV infection may play an important role in the development of PVTT [15]. The potential correlation between HBV replication and the formation of MVI in HCC have also been studied in some preliminary clinical studies. Chen et al. retrospectively studied the impact of ascites, as well as tumor- and HBV-related factors, on the formation of vascular invasion and found negative results concerning the impact of viral factors; however, it is worth noting that the limited number of cases with MVI (*n* = 12) and incomplete data concerning the status of HBV infection may limit the power of their results [21]. To establish a preoperative prediction model for MVI, a large cohort of HBV-related HCC patients (*n* = 1004) was analyzed by Lei et al., revealing that a high seral HBV DNA load (> 10^4^ IU/ml) was an independent factor predicting the presence of MVI. The other predictive variables were well-known tumor-related factors, including a large tumor diameter, multiple nodules, an incomplete capsule and an AFP level > 20 ng/ml [16]. Nevertheless, the relationship between HBV infection and vascular invasion has rarely been intentionally researched in a well-designed clinical study.

Our study was based on a prospectively collected database with comprehensive data indicating the status of HBV infection and vascular invasion. The results showed that compared with patients without HBV infection, the incidence of vascular invasion, including MVI and PVTT, was significantly increased in HBV-related HCC. In the multivariate analysis carried out in positive HBsAg patients, positive HBeAg and a detectable seral HBV DNA load (> 50 IU/ml) were significantly associated with development of vascular invasion. In addition, our results revealed that in HBV-related HCC patients, a more severe level of vascular invasion was associated with a higher rate of active HBV replication, as reflected in positive HBeAg or a detectable HBV DNA load. These findings provided promising clinical evidence to demonstrate that in addition to tumor-related factors, the activity of HBV infection plays a key role in the development of vascular invasion in HCC patients.

The postoperative recurrence of HBV-related HCC was categorized into two groups, early and late recurrence, with a cut-off time at 2 years [22]. Late recurrence (> 2 years after resection) usually presented as a metachronous tumor with different genetic and histological features from the primary HCC [22, 23]. It was revealed that HBV-related factors, including a high hepatic inflammatory activity score and high HBV DNA load, were significantly associated with late recurrence, whereas sustained suppression of HBV replication by anti-viral drugs achieved a lower rate of late recurrence [12, 24, 25]. Tumors occurring within 2 years after surgery were classified as early recurrence, which was strongly associated with tumor-related factors, including tumor size and the presence of nodules, vascular invasion and resection margin [22]. A randomized controlled trial by Lin et al. revealed that patients receiving anti-viral treatment showed a significantly better 2-year overall (93.8% vs 62.2%) and recurrence-free (55.6% vs 19.5%) survival [26]. However, it is difficult to understand the effect of anti-viral drugs on inhibiting early postoperative recurrence (< 2 years after resection), which was considered the result of the regrowth of micro-metastases in the liver that were not detected and resected during the operation. Our result demonstrates that active HBV replication is associated with a high rate of vascular invasion in HCC patients, which may partially explain the anti-tumor effect of antiviral treatment. We could speculate that the suppression of HBV replication via anti-viral treatment might decrease the invasiveness and metastatic potential of HCC to reduce the risk of early postoperative recurrence.

The seral HBV DNA load is usually divided into high and low levels at a cut-off value of 2000 IU/ml. In this study, the impact of the seral HBV DNA load on the formation of vascular invasion was not significant if the cut-off value was set at this point. This result implies that the correlation between the HBV DNA load and occurrence of vascular invasion is not linear. Additionally, this result may also be caused by a proportion of patients with a high HBV DNA load being in the “immune tolerant” phase with no or mild substantial liver injury [10]. According to the current guidelines, anti-viral drugs should be prescribed in chronic hepatitis B patients with a serum HBV DNA load above 2000 IU/ml and elevated ALT levels, in the absence of sufficient evidence of cirrhosis [10]. However, for patients with a low HBV DNA level (< 2000 IU/ml) without advanced liver disease, the benefit of anti-viral treatment has not been well clarified. In this study, HCC patients with an undetectable HBV DNA load (≤ 50 IU/ml) had a lower incidence of vascular invasion than patients with a detectable HBV DNA load (> 50 IU/ml). These results suggested that it may also be beneficial to receive anti-viral drugs for patients who do not meet the current treatment indication.

The suppressed HBV replication by anti-viral treatment was supposed to correlate with a lower rate of vascular invasion. However, the inhibitory effect of anti-viral treatment on the development of vascular invasion was overshadowed by tumor-related factors in the multivariate analysis. The following reasons may explain this phenomenon. First, patients who have undergone anti-viral treatment usually have no or mild cirrhosis with normal liver function [27, 28]. Surgeons are more likely to apply hepatectomy with a wider resection margin to these patients. Theoretically, a wide surgical margin will lead to a higher detection rate of MVI during pathological evaluation. Second, patients who have undergone anti-viral treatment have enjoyed good health care and regular surveillance, leading to early detection of HCC. Thus, the inhibitory effect of anti-viral treatment tended to be overshadowed by the early tumor features.

It is worth noting that MVI and PVTT have different risk factor profiles in our research. For HBV-related factors, only detectable HBV load was associated MVI, while only positive HBeAg was associated with PVTT. Although MVI and PVTT were two common types of vascular invasion of HCC, there was no evidence indicating potential causal relationship between them. Previous clinic studies also revealed that MVI and PVTT had inconsistent predicting factors [2, 5, 7, 8]. Further fundamental and clinic studies are needed to clarify the relationship between MVI and PVTT.

This study might not be able to reveal the full landscape of the relationship between HBV activity and the occurrence of vascular invasion in HCC. In particular, because of the limited follow-up time, we failed to carry out a survival analysis to determine significant factors contributing to recurrence or survival. However, the main finding of this study is the association between HBV infection status and presence of vascular invasion in HCC, lack of survival information may have a less impact on our conclusion. Additionally, inconsistency existed between the protocol of anti-viral treatment and surgical procedures because of the retrospective nature of this research. Furthermore, the surgical margin varied in patients with different levels of cirrhosis, a finding that might affect the detection rate of MVI. At last, only HBV-related HCC was studied in this research, the conclusion isn’t applicable for HCC caused by other hepatic virus. Despite these limitations, we first found the interesting phenomenon that HBV infection and replication status were independently associated with the formation of vascular invasion in HCC, which may partially explain the inhibitory effect of anti-viral treatment on early HCC recurrence.

## Conclusions

In addition to characteristics of the tumor itself, HBV infection and active replication were independently associated with the development of vascular invasion in HCC. In patients with HBV-related HCC with positive HBeAg or a detectable HBV DNA load, an increased risk of vascular invasion should be recognized.

## Abbreviations

AFP: α-fetoprotein
DCP: Desc-carboxy Prothrombin
HBeAg: Hepatitis B e-antigen
HBsAg: Hepatitis B surface-antigen
HBV: Hepatitis B Virus
HBx: HBV X Protein
HCC: Hepatocellular Carcinoma
HCV: Hepatitis C Virus
MVI: Microvascular Invasion
PVTT: Portal Vein Tumor Thrombus
TACE: Transarterial Chemoembolization
